# A Case of White Swelling Cured by Iodine

**Published:** 1844-10

**Authors:** John P. Anderson

**Affiliations:** Sparta Alabama


Art. II.—*A Case of White Swelling cured by Iodine.* By Dr. John P. Anderson, of Sparta, Ala.

I propose communicating the particulars of a case of *white swelling* successfully treated with Iodine, which, if you think it will be of any service to the profession, you may give a place in your journal.

Miss W. was attacked at the age of seven with white swelling just above the knee. An eminent physician was called to see her, and succeeded in affording her temporary relief. Some months thus elapsed, when the symptoms became more aggravated, and medical aid was again sought. She was a second time partially relieved for a few months, but the symptoms then returning, her case was abandoned as irremediable before the age of puberty. The parents became alarmed at this annunciation, and betook themselves to every nostrum that could be heard of, from which nothing resulted to the patient but the most extreme torture. In this manner things progressed for twelve years, at which time she presented herself to me an object of pity, soliciting my efforts to effect a cure. After hearing the history of her case I consented to undertake it, but without much promise of success. I commenced by correcting the morbid condition of her digestive organs, which was very decided. For this, a few mercurial cathartics, with a regulated diet, were found sufficient.

The limb on examination proved to be very much swelled, and presented three ugly ulcers, which discharged freely a thin curdled pus. I directed the patient to take tincture of iodine in doses of 15 drops three times daily, increasing the dose to 30 drops three times a day. The medicine then to be suspended for ten days, and resumed in doses of 20 drops, to be increased to 40 drops—again to be suspended for 10 days, and then resumed, as before. In this way I kept it up for eighteen months, never giving less than 20 drops, nor more than 40, and never suspending it for a longer time than fifteen days. No other medicine was administered internally, except an occasional dose of castor oil. At the commencement I applied a bandage to the limb which very soon reduced it to its natural size; the ulcers were kept clean with warm water. The limb having been relieved in a great measure of swelling, I applied a plaster over it which had the effect of protecting the ulcers at once from the meddling of the patient, and the action of the atmosphere. I am indebted to a 'knowing old woman' for the formula, and give it in the language in which I received it from her:

℞ Red precipitate,
Red lead,
Spanish brown,
Spirits Turpentine,
British oil, each ℥iij.
Soft rosin, lb$\frac{3}{4}$.

The ingredients are to be well mixed, and spread upon soft buckskin. To be kept on eight or ten days at a time.

In the course of six weeks numerous spiculæ of the bone were expelled, and at every subsequent dressing for a long time others appeared. At the end of sixteen months the two ulcers highest on the limb had entirely healed, leaving one on the outside of the knee joint, in which was a large spicula of bone appearing to adhere to the bottom of the ulcer, and projecting above its surface. Finding that it could not be extracted without great pain, I cut off the protruded part with a pair of pliers. At the next dressing I had to repeat the clipping, when it appeared to have become entirely detached, but was too large to be removed without enlarging the orifice considerably. I suffered it to remain ten days as it now gave no pain, at which time I enlarged the orifice and removed a triangular shaped, slightly curved piece of bone, measuring nearly two inches in its greatest diameter, and $\frac{1}{16}$ of an inch in thickness, of a spongy texture. The orifice healed kindly in a short time, leaving the joint anchylosed, the patella fixed, and the limb considerably shortened. The medicine and plaster were now discontinued, a rosy cheek had taken the place of the sallow complexion, the whole frame had assumed the appearance peculiar to her sex, the crutch which had been a twelve years' burden was laid aside, and no symptom has threatened a return of the disease since my patient was discharged, which is now three and a half years.

Sparta, Ala., July, 1844.

